# Stage III deficient mismatch repair colon patients get greater benefit from earlier starting oxaliplatin-based chemotherapy regimen

**DOI:** 10.1038/s41598-023-33153-8

**Published:** 2023-06-02

**Authors:** Yixiang Zhan, Kemin Ni, Zhaoce Liu, Ran xin, Qiurong Han, Hangyu Ping, Yaohong Liu, Xuanzhu Zhao, Wanting Wang, Suying Yan, Jing Sun, Qinghuai Zhang, Guihua Wang, Zili Zhang, Xipeng Zhang, Xia Hu, Guoxun Li, Chunze Zhang

**Affiliations:** 1 Department of Colorectal Surgery, Tianjin Union Medical Center, Tianjin, 300121 China; 2 School of Medicine, Nankai University, Tianjin, China; 3 Tianjin Institute of Coloproctology, Tianjin, China; 4 School of Integrative Medicine, Tianjin University of Traditional Chinese Medicine, Tianjin, China; 5 The Institute of Translational Medicine, Tianjin Union Medical Center of Nankai University, Tianjin, China; 6 Tongji Hospital, Tongji Medical College, Huazhong University of Science and Technology, Wuhan, China; 7 The Third Central, Clinical College of Tianjin Medical University, Tianjin, China; 8 Department of Agriculture Insect, Institute of Plant Protection, Tianjin Academy of Agricultural Sciences, Tianjin, China

**Keywords:** Gastrointestinal cancer, Surgical oncology

## Abstract

We evaluate the prognostic value of chemotherapy and other prognostic factors on overall survival among colon patients with deficient mismatch repair (dMMR), and determine the optimum time to start chemotherapy after surgery. Data of 306 colon cancer patients with dMMR who received radical surgery were collected from three Chinese centers between August 2012 and January 2018. Overall survival (OS) was assessed with the Kaplan–Meier method and log-rank. Cox regression analysis were used to assess influencing prognosis factors. The median follow-up time for all patients was 45.0 months (range, 1.0–100). There was a nonsignificant OS benefit from chemotherapy for patients with stage I and stage II disease, including high-risk stage II disease (log-rank* p*: 0.386, 0.779, 0.921), and a significant OS benefit for patients with stage III and stage IV disease for receiving post-operation chemotherapy (log-rank *p* = 0.002, 0.019). Stage III patients benefitted from chemotherapy regimens that contained oxaliplatin (log-rank *p* = 0.004), and Starting chemotherapy with oxaliplatin treatment earlier resulted in better outcomes (95% CI 0.013–0.857; *p* = 0.035). Chemotherapy regimens containing oxaliplatin can prolong the survival time of stage III and IV dMMR colon cancer patients. This beneficial manifestation was more pronounced after starting chemotherapy treatment early post operation. High risk stage II dMMR colon patients including T_4_N_0_M_0_ cannot benefit from chemotherapy.

## Introduction

Colorectal cancer (CRC) is the third most commonly diagnosed cancer, and its mortality is the fourth highest worldwide. CRC is a heterogeneous disease due to its different clinical manifestations and etiologies^1^.

Most newly diagnosed CRC patients can undergo a thorough operation to remove primary tumors through laparoscopic-assisted or open surgery, resulting in long disease-free survival^2^. However, many postoperative patients develop local or remote relapse, and patients at a high risk of relapse are recommended to receive adjuvant chemotherapy treatment, as shown in a variety of previous studies^3,4^. It is necessary to select such patients through various methods and provide further treatment before cancer recurrence. Many biomarkers have been used to determine whether chemotherapy is needed or not^5^. For the past several years, a consensus has emerged among medical workers that the state of mismatch repair (MMR) could be used as a critical biomarker for mid-term and advanced CRCs to guide postoperative treatment^6,7^.dMMR can give rise to the activation of proto-oncogenes or the inactivation of anti-oncogenes. Approximately 10–20% of sporadic CRC show MMR deficiency by immunohistochemistry (IHC), as shown in many previous studies^5,8–10^. Among colon cancers, dMMR and proficient MMR (pMMR) colon cancers behave differently in many aspects, such as sensitivity to chemotherapy, clinicopathological features, pathogenesis and so on^11^. However, the optimal treatment for early-stage disease is controversial. A lot of studies have proved that MMR not only accelerates the progression of malignant tumors but also plays a crucial role in the prognosis and relapse of tumors^12,13^. CRC with dMMR with infiltration by cytotoxic T cells and natural killer cells, which can inhibit tumor cell growth and results in a better prognosis, is associated with a longer patient survival time than pMMR CRC^14,15^. Unfortunately, the isolated impact of the MMR status on stage II colon cancer patients has not been evaluated.

The MMR system is activated after the MutS protein homolog 2 (MSH2)/ MutS protein homolog 6 (MSH6) heterodimer combines with DNA mismatch base pairs, followed by interaction with the MutL homolog 1 (MLH1)/ PMS1 homolog 2 (PMS2) heterodimer, which acts as a sliding clamp structure, and ultimately the deletion of mismatch base pairs by exonuclease 1 (EXO1)^16^. IHC is used frequently to recognize the dMMR proteins because this method does not need the complicated instruments that PCR requires and has the ability to identify mutated genes that code MMR proteins. In former studies, there were no significant differences between using immunohistochemistry and genotyping separately^17^.

According to prior studies, pathological characteristics are also associated with tumor prognosis. Among all characteristics, cancer site, T stage and lymphatic metastasis are the major factors that influence prognosis^18^. The identification of patients at high risk of recurrence is crucial for selecting candidates for adjuvant chemotherapy. Although many previous studies have attempted to expound the relationship between MMR types and prognoses among CRC patients, which mixed receiving chemotherapy post-operation and excision merely patients^19^. Additionally, there are few studies on individuals of Asian ethnicity alone^18^. Because of these shortcomings, we designed a new study in which dMMR colon cancer patients were strictly selected to determine the impact of adjuvant chemotherapy among these populations. It would be significant to accurately identify the survival differences among the subgroups due to chemotherapeutic agents.

In this study, we aimed to evaluate the prognostic value of chemotherapy and other prognostic factors on OS among colon patients with dMMR using data from multicenter. At the same time, we also aimed to determine the optimum time to start chemotherapy after surgery.

## Method

### Study population

All consecutive patients included in this study had received curative surgery for colon cancer between August 2012 and January 2018 in three Chinese centers (Tianjin Union Medical Center, The Third Central Clinical College of Tianjin Medical University, Tongji Hospital). These patients had pathological reports and were diagnosed with dMMR colon cancer at the hospital pathology centers, and some had received chemotherapy after surgery as recommended by the physicians. Stratification factors included the following: age, number of metastatic lymph nodes, tumor location, histologic grade, and T stage. We included unstated grades, special type adenocarcinoma and non-adenocarcinoma into the group named special grade.

The inclusion criteria were as follows: 1. age more than 18 years, 2. histological confirmation of colon cancer and dMMR, 3. follow-up time more than 3 years, and 4. adequate demographics and follow-up treatment data. There were no special exclusion criteria.

Patient pathology and baseline data were obtained using the hospital data system. Patient survival data were obtained from medical records, phone call consultations and local health care administrations. Every patient was censored in February 2021.

### Determination of the MMR status

The MMR tumor status was determined by IHC. dMMR tumors were defined as those with loss of the expression of one or more MMR proteins. MLH1, MutS protein homolog 2 (MSH2), MutS homolog 6 (MSH6) and PMS2 are the main proteins involved in the MMR system. MMR protein expression was analyzed by immunohistochemistry in formalin-fixed, paraffin-embedded tumor sections^20^. If at least one of the MMR proteins was not expressed, the patient was allocated to the dMMR group. Each section was judged by two pathologists.

### Statistical analysis

The primary outcome was OS, assessed with the Kaplan–Meier method. OS time was measured from the date of excision to the date of death from any cause. Classified variables and disorder data were calculated using the Chi-Squared test. Continuous variables were calculated using the t-test. Wilcoxon rank sum test was used to analyze the differences among these ranked data. A two-sided P value less than 0.05 was considered statistically significant. All statistical analyses were performed with SPSS software version 23 (SPSS Inc., Chicago, IL, USA).

### Ethics approval and consent to participate

This study was approved by the Ethics Committee of Tianjin Union Medical Center. The need for informed consent was waived by the ethics committee of Tianjin Union Medical Center. All methods were carried out in accordance with relevant guidelines and regulations.

## Results

### Patient characteristics

**Figure 1:**
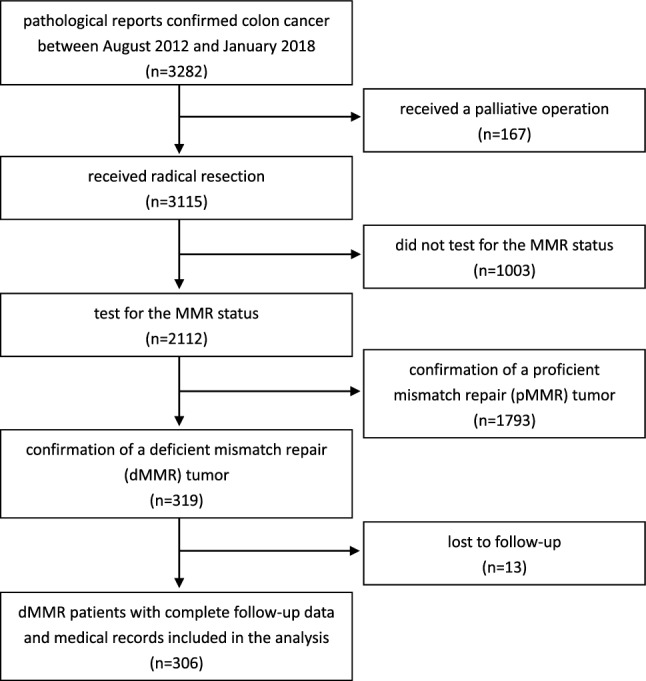
Flow diagram of the study population.

A total of 306 individuals diagnosed with colon cancer with dMMR between August 2012 and January 2018 were included in this study. A diagram depicting the complete selection process is shown in Fig. 1. The median follow-up time for all patients was 45.0 months (range, 1.0–100). All patients received radical resection, and the dMMR status was determined by IHC. In total, 165 patients received adjuvant chemotherapy after surgery, including 98 (59.4%) patients who received the mFOLFOX6 regimen, 30 (18.2%) patients who received capecitabine, 19 (11.5%) patients who received the FOLFIRI regimen, and 18 (10.9%) patients who received the XELOX regimen.

Overall, the median age at diagnosis was 63 years (range, 21–87 years); 57% were males who accepted adjuvant chemotherapy, and patients with TNM stage II disease accounted for the vast majority (from stages I to IV, 8.2%, 63.1%, 24.5%, and 4.2%). We classified mucinous adenocarcinoma separately for the statistical analysis because patients with dMMR tend to be diagnosed with mucinous adenocarcinoma. Baseline characteristics of patients in the chemotherapy arm and surgery arm are shown in Table 1.

**Table 1:** Demographic and clinical characteristics of all dMMR patients.

	Total (No. %)	Surgery alone group (No. %)	Chemotherapy group (No. %)	*p*
Sex				0.032
Male	157 (51.3%)	63 (44.7%)	94 (57.0%)	
Female	149 (48.7%)	78 (55.3%)	71 (43.0%)	
Age				0.087
< 60	120 (39.2%)	48 (34.0%)	72 (43.6%)	
≥ 60	186 (60.8%)	93 (66.0%)	93 (56.4%)	
Depth of tumor invasion (T)				0.393
T1	12 (3.9%)	8 (5.7%)	4 (2.4%)	
T2	15 (4.9%)	7 (5.0%)	8 (4.8%)	
T3	211 (69.0%)	92 (65.2%)	119 (72.1%)	
T4	68 (22.2%)	34 (24.1%)	34 (20.6%)	
Positive lymph nodes (N)				0.867
0	219 (71.5%)	102 (72.3%)	117 (70.9%)	
1–3	58 (19.0%)	25 (17.7%)	33 (20.0%)	
≥ 4	29 (9.5%)	14 (9.9%)	15 (9.1%)	
TNM				0.601
1	25 (8.2%)	15 (10.6%)	10 (6.1%)	
2	193 (63.1%)	86 (61.0%)	107 (64.8%)	
3	75 (24.5%)	33 (23.4%)	42 (25.5%)	
4	13 (4.2%)	7 (5.0%)	6 (3.6%)	
Histologic grade				0.820
Low (1/2)	146 (47.7%)	70 (49.6%)	76 (46.1%)	
High (3/4)	110 (35.9%)	49 (34.8%)	61 (37.0%)	
Special type	50 (16.3%)	22 (15.6%)	28 (17.0%)	
Location				0.024
Proximal colon	208 (68.0%)	105 (74.5%)	103 (62.4%)	
Distal colon	99 (32.0%)	36 (25.5%)	63 (37.6%)	
Operation				0.432
Open surgery	219 (71.6%)	104 (73.8%)	115 (69.7%)	
Laparoscopic	87 (28.4%)	37 (26.2%)	50 (30.3%)	
Total	306 (100%)	141 (46.1%)	165 (53.9%)	

### Overall survival and adjuvant chemotherapy in the whole population

Of the whole population, 61 patients died during the follow-up period. There was 1 OS event in a patient with stage I disease, 20 OS events in patient with stage II disease, 27 OS events in patients with stage III disease and 13 OS events in patients with stage IV disease, resulting in OS rates of 96.0%, 89.6%, 64.0%, and 0%, respectively.

**Figure 2:**
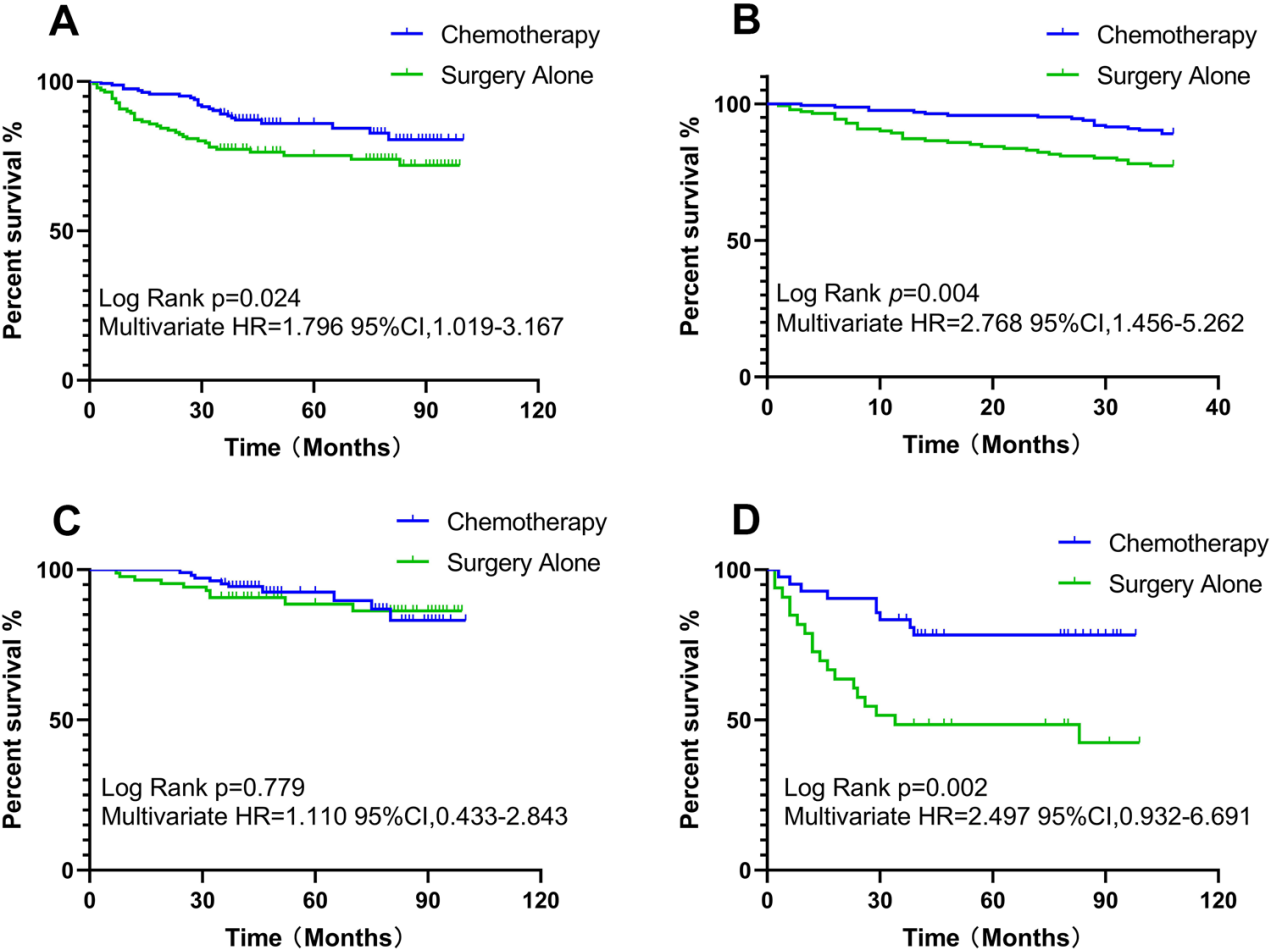
Kaplan–Meier curve of overall survival. (**A**) comparing surgery alone with chemotherapy for all patients; (**B**) 3-year overall survival comparing surgery alone with chemotherapy for all patients; (**C**) comparing surgery alone with chemotherapy for stage II patients; (**D**) comparing surgery alone with chemotherapy for stage III patients.

The overall OS rate was 74.5% for patients treated with surgery alone and 84.8% for patients treated with adjuvant chemotherapy. This difference was statistically significant (log-rank *p* = 0.024) in the Kaplan–Meier curve of OS. Multivariate Cox regression analysis revealed that the benefit of surgery alone was significantly worse than that of chemotherapy (HR, 1.796; 95% CI 1.019–3.167, *p* = 0.043). There were nonsignificant OS benefits for patients with stage I, stage II and high-risk stage II disease (log-rank *p* = 0.386, 0.779, 0.921) and significant OS benefits for patients with stage III and stage IV disease (log-rank *p* = 0.002, 0.019, Fig. 2).

**Figure 3:**
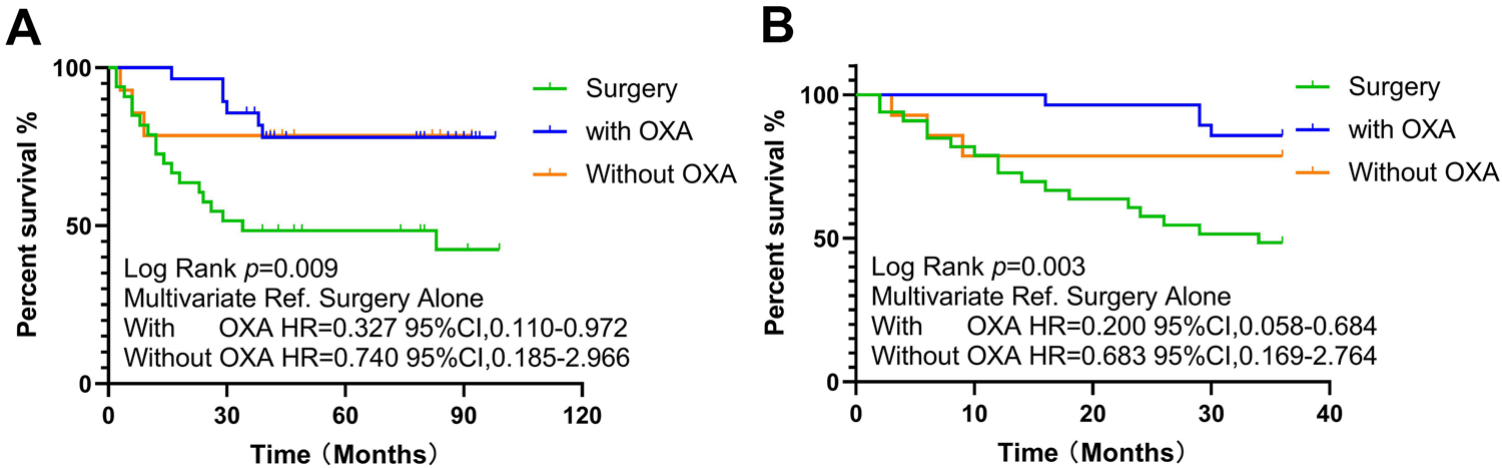
Kaplan–Meier curve of overall survival for stage III patients stratified by adjuvant chemotherapy regimens. (**A**) Overall survival; (**B**) 3-year overall survival.

Furthermore, we evaluated the effect of chemotherapy with or without oxaliplatin on OS for stage III patients. Adjuvant chemotherapy with or without oxaliplatin had different prognostic effects on these patients. Patients achieved an OS benefit from chemotherapy regimens that contained oxaliplatin (contain oxaliplatin vs. surgery alone; log-rank *p* = 0.004, Fig. 3). This benefit was also observed with surgery alone according to the multivariate Cox regression analysis (HR, 0.327; 95% CI 0.110–0.972; *p* = 0.044). This benefit was not verified in the arm without oxaliplatin (log-rank *p* = 0.087) or in patients with high-risk stage II disease (log-rank *p* = 0.767). Regrettably, this study did not analyze enough stage IV patients to verify this conclusion. We specifically analyzed T_4_N_0_M_0_ patients and found that they did not benefit from chemotherapy (log-rank *p* = 0.264).

### Timing of chemotherapy initiation and prognosis in stage III patients

We divided the experimental group into two groups, the early intervention group (within 28 days of surgery) and the late intervention group (after 28 days), according to the time of initiation of chemotherapy containing oxaliplatin.

The OS rate for patients in the early intervention group was 93.8 and that for patients in the late intervention group was 58.3%. The multivariate HRs for these two groups compared with the surgery alone group were 0.106 (95% CI 0.013–0.857; *p* = 0.035) and 0.584 (95% CI 0.181–1.888; *p* = 0.369, Table 2), respectively. However, the univariate HR for OS of the early intervention group compared with the late intervention group was 7.884 (95% CI 0.918–67.681; *p* = 0.060).

**Table 2:** Univariate and multivariate Cox regression models for OS in stage III patients.

Variable	Univariate analysis		Number	Multivariate analysis	
	HR (95% CI)	*P*		HR (95% CI)	*p*
Sex					
Female	1	0.633	37		
Male	1.203 (0.563–2.572)		38		
Age	1.077 (1.036–1.118)	< 0.001	75	1.048 (1.004–1.094)	0.030
Depth of tumor invasion (T)					
T1–T3	1	0.451	56		
T4	1.376 (0.600–3.156)		19		
Positive lymph nodes (N)					
1–3	1	0.060	53		
≥ 4	2.076 (0.969–4.445)		22		
Location					
Proximal colon	1	0.923	48		
Distal colon	1.039 (0.476–2.271)		27		
Operation					
Open surgery	1	0.089	51		
Laparoscopic	0.430 (0.163–1.136)		24		
Positive lymph nodes	1.165 (1.058–1.284)	0.002	75	1.167 (1.048–1.300)	0.005
Histologic grade					
Low grade (1/2)	1		27	1	
High grade (3/4)	2.870 (1.136–7.252)	0.026	37	2.908 (1.115–7.585)	0.029
Special type	1.292 (0.323–5.176)	0.717	11	0.967 (0.218–4.298)	0.965
Chemotherapy regimens					
Surgery alone	1		33	1	
Oxaliplatin early	0.082 (0.011–0.613)	0.015	16	0.106 (0.013–0.857)	0.035
Oxaliplatin later	0.594 (0.220–1.603)	0.304	12	0.584 (0.181–1.888)	0.369
Other regimens	0.338 (0.099–1.151)	0.083	14	0.758 (0.188–3.059)	0.697

In the meantime, we compared the OS of patients treated with oxaliplatin-based chemotherapy with those treated with FU alone (including infusion 5–FU and oral capecitabine). Curiously and counter intuitively, the early or late intervention oxaliplatin-based group do not have obvious benefit than 5-FU alone group (95% CI 0.576–6.652 *p* = 0.281; 95% CI 0.017–1.537 *p* = 0.112, respectively).

No association was discovered between dMMR and N stage (HR = 2.076; 95% CI 0.969–4.445; *p* = 0.060) or T stage (HR = 1.376; 95% CI 0.600–3.156; *p* = 0.362). Nevertheless, stage III with dMMR colon cancer had different prognostic effects depending on the number of positive lymph nodes detected during surgery.

## Discussion

MMR was the first genetic biomarker that was used to guide the selection of adjuvant treatment after surgery, and it is generally believed that dMMR colon cancer, which is commonly located in the proximal colon, has a better prognosis than pMMR colon cancer^21,22^. The current NCCN guidelines recommend that all CRC patients undergo MMR status testing through IHC or PCR^23^. However, for high-risk stage II colon cancer treated with fluoropyrimidine alone, there is still a matter of debate about the optimal chemotherapy regimen^24,25^. Some reports have demonstrated the no effectiveness of chemotherapy regimens, and others have demonstrated a deleterious effect of fluoropyrimidine among patients with stage II dMMR CRC^8,26^. In this study, we strictly specified standards in order to guarantee that all selected patients had dMMR colon cancer of all TNM stages. And our multicenter data is more representative.

In the total population, receiving chemotherapy was associated with better outcomes than undergoing surgery lonely (HR = 1.783; 95% CI 1.070–2.973; *p* = 0.027). The latest China cancer data shows that most deaths occur after the age of 60, and the median age at diagnosis was 63 years in our study. So, we divided the patients into groups based on whether they were over 60 years old. Table 1 shows that distal colon cancer was significantly more prevalent in the chemotherapy group than in the surgery alone group, but there was no significant association between patient survival time and location of the dMMR cancer (proximal cancer vs. distal cancer: HR = 0.899; 95% CI 0.519–1.559; *p* = 0.705). After analyzing each TNM stage, this association was found to exist only in stage III (HR = 3.281; 95% CI 1.469–7.326; *p* = 0.004) and stage IV (HR = 5.130; 95% CI 1.270–20.715; *p* = 0.022).

We separately analyzed the effect of chemotherapy on high-risk stage II patients but still did not find an association between prognosis and chemotherapy (HR = 1.062; 95% CI 0.323–3.494; *p* = 0.921). Our study proved T_4_N_0_M_0_ dMMR patients could not benefit from chemotherapy.

Here, we decided to divide the included stage III patients receiving chemotherapy into two groups according to whether the regimens included oxaliplatin. Further analysis revealed that patients benefited more from chemotherapy regimens that included oxaliplatin (HR = 0.290; 95% CI 0.115–0.734; *p* = 0.009) versus those that did not include oxaliplatin (HR = 0.338; 95% CI 0.099–1.152; *p* = 0.083). Some previous findings have shown that the chemotherapy sensitivity to oxaliplatin is independent of the MMR system, given that oxaliplatin-DNA adducts, a distortion of the secondary DNA structure, cannot be distinguished by MMR-induced responses^27–29^. In this study, we only explored whether dMMR patients can get benefit from different chemotherapy regimens. The further study of National Surgical Adjuvant Breast and Bowel Project (NSABP) C-07 and C-08 found that colon cancer patients can get benefit from oxaliplatin independent of the MMR status^30^. In our published research, we get the similar conlusion for both dMMR and pMMR patients^31^.

Furthermore, we observed that patient outcomes were related to the time at which chemotherapy was started. We divided stage III dMMR colon cancer patients receiving chemotherapy containing oxaliplatin into two groups based on the median start day after surgery (28 days). Patients who started chemotherapy early (within 28 days of surgery) benefited significantly more from chemotherapy than those who received surgery alone (HR = 0.290; 95% CI 0.115–0.734; *p* = 0.009), and this finding was not observed in patients who started chemotherapy late (after 28 days). The most likely reason for this phenomenon is that only the “base excision repair” system responded to DNA damage with dMMR^32^. Moreover, 5-FU can lead to the accumulation of DNA damage targeted by another repair pathway^33^. This benefit was also verified in the 3-year OS outcome of stage III dMMR colon cancer patients (HR = 0.088; 95% CI 0.012–0.662; *p* = 0.018). These data included only 10 patients who were treated with FU alone, which the guide does not recommend using this treatment for stage III patients. The phenomenon that the early or late intervention oxaliplatin-based group do not have obvious benefit than 5-FU alone group in stage III patients may be caused by the small number of people treated with 5-FU alone, which is under-representation.

Old age, poor differentiation, more positive lymph nodes detected, advanced TNM stage, and surgery alone were independently associated with poor outcomes in all dMMR patients. Most of the OS events occurred within three years after radical surgery (OS, 80.1%; 3-year OS, 83.4%). This finding is similar to that of the study by Romain Cohen^34^. By analyzing only TNM stage III dMMR colon cancer patients, we concluded that the significant prognostic risk factors were old age, positive lymph nodes detected, poor differentiation, and surgery alone. Chemotherapy without oxaliplatin (HR = 0.740; 95% CI 0.185–2.966; *p* = 0.671) and T stage (HR = 1.376; 95% CI 0.600–3.156; *p* = 0.362) had nonsignificant correlations with prognosis. Interestingly, we did not find that N stage was significantly associated with prognosis, although this association with the number of lymph nodes detected has been confirmed in whole colon cancer patient’s cohort. Some previous studies have shown that the number of lymph node metastases in predicting prognosis is related to the primary tumor location and MMR status^35–37^. This result suggests that the same number of lymph node metastases may have different levels of significance for different MMR states. Regrettably, due to the limited number of people included in this study, we were unable to demonstrate this impact.

Limitations to this study include the methods used to detect the expression of MMR proteins. The accuracy of IHC is dependent on the pathologist’s experience and is lower than that of PCR and high-throughput sequencing^38^. Secondly, we did not obtain the BRAF and RAS statuses of these patients, which made it impossible to analyze the impact of these mutations on the prognosis of dMMR patients^39^. In addition, this is a retrospective investigation and therefore impacted by extrinsic factors such as patient performance status that might necessitate delays in treatment or push providers to be more or less inclined to pursue oxaliplatin-based chemotherapy. The absence of a multifactorial analysis of these factors may have influenced the results.

In conclusion, this retrospective study suggests that patients with dMMR colon cancer, especially stage III colon cancer, can get benefit from chemotherapy containing oxaliplatin, and this chemotherapy regimen should be started sooner rather than later. High risk stage II dMMR colon patients including T_4_N_0_M_0_ cannot benefit from chemotherapy. Additionally, we should pay close attention to the role of the number of positive lymph nodes detected in the stratification of patients.

## Data Availability

The datasets used and analyzed during the current study are available from the corresponding author on reasonable request.

## Abbreviations

OS: Overall survival
MMR: Mismatch repair
dMMR: Deficient mismatch repair
pMMR: Proficient mismatch repair
IHC: Immunohistochemical
EXO1: Exonuclease 1
MLH1: MutL homolog 1
MSH2: MutS protein homolog 2
MSH6: MutS homolog 6
PMS2: PMS1 homolog 2
